# Lifelong Engagement: The Role of Cognitive Reserve and Physical Health in Very Late Life

**DOI:** 10.1093/geroni/igaa057.2820

**Published:** 2020-12-16

**Authors:** Peter Martin, Bradley Willcox, D Craig Willcox

## Abstract

At the end of a very long life, older adults often experience a significant decline in cognitive function. However, there are older adults who have maintained high levels of cognition and physical health. The purpose of this symposium is to illuminate interdisciplinary findings of cognitive engagement with late-life benefits of cognitive functioning and physical health. Components of cognitive reserve include sociodemographic variables (e.g., education, occupational complexity and responsibility), psychosocial variables (e.g., engaged life style and activity) and physical and genetic reserve (e.g., strength, APOE4). Based on three major research studies (the Japanese SONIC study; the Honolulu Asia Aging Study, HAAS; and the Georgia Centenarian Study, GCS), we highlight important aspects of building cognitive reserve and the implications for cognitive and physical health. The first presentation evaluates the importance of work complexity as a predictor of cognitive and physical health among participants of the SONIC study. Multiple group analyses yielded strong associations of occupational complexity with cognitive functioning for men. The second presentation reports logistic regression findings from the HAAS including education, strength and genetic markers, as well as mental health and their relatedness to cognitive abilities and physical health. The final presentation evaluates a structural equation model from the GCS, highlighting the interrelationship of cognitive reserve components (i.e., education, occupational responsibility, engaged lifestyle, social support, and activity) with cognitive and physical health in very late life. We will summarize and integrate the findings for their theoretical and practical implications and provide future directions.

